# Detecting paroxysmal atrial fibrillation from normal sinus rhythm in equine athletes using Symmetric Projection Attractor Reconstruction and machine learning

**DOI:** 10.1016/j.cvdhj.2022.02.001

**Published:** 2022-02-14

**Authors:** Ying H. Huang, Jane V. Lyle, Anisa Shahira Ab Razak, Manasi Nandi, Celia M. Marr, Christopher L.-H. Huang, Philip J. Aston, Kamalan Jeevaratnam

**Affiliations:** ∗ Faculty of Health and Medical Sciences, University of Surrey, Guildford, United Kingdom; † Department of Mathematics, University of Surrey, Guildford, United Kingdom; ‡ Department of Veterinary Medicine, University of Cambridge, Madingley Rd, Cambridge, United Kingdom; § School of Cancer and Pharmaceutical Sciences, Faculty of Life Sciences & Medicine, King's College London, London, United Kingdom; ‖ Rossdales Equine Hospital and Diagnostic Centre, Newmarket, United Kingdom; ¶ Physiological Laboratory, University of Cambridge, Cambridge, United Kingdom

**Keywords:** Paroxysmal atrial fibrillation, Symmetric Projection Attractor Reconstruction, Equine ECG signals, Normal sinus rhythm, Diagnostic, Machine learning

## Abstract

**Background:**

Atrial fibrillation (AF) is a common cardiac arrhythmia in both human and equine populations. It is associated with adverse outcomes in humans and decreased athletic performance in both populations. Paroxysmal atrial fibrillation (PAF) presents with intermittent, self-terminating AF episodes, and is difficult to diagnose once sinus rhythm resumes.

**Objective:**

We aimed to detect PAF subjects from normal sinus rhythm equine electrocardiograms (ECGs) using the Symmetric Projection Attractor Reconstruction (SPAR) method to encapsulate the waveform morphology and variability as the basis of a machine learning classification.

**Methods:**

We obtained ECG signals from 139 active equine athletes (120 control, 19 with a PAF diagnosis). The SPAR method was applied to 9 short (20-second) ECG strips for each subject. An optimal SPAR feature set was determined by forward feature selection for input to a machine learning model ensemble of 3 different classifiers (*k*-nearest neighbors, linear support vector machine, and radial basis function kernel support vector machine). Imbalanced data were handled by upsampling the minority (PAF) class. A final subject classification was made by taking a majority vote over results from the 9 ECG strips.

**Results:**

Our final cross-validated classification for a subject gave an accuracy of 89.0%, sensitivity of 94.8%, specificity of 87.1%, and receiver operating characteristic area under the curve of 0.98, taking PAF as the positive class.

**Conclusion:**

The SPAR method and machine learning generated a final model with high sensitivity, suggesting that PAF can be discriminated from short equine ECG strips. This preliminary study indicated that SPAR analysis of human ECG could support patient monitoring, risk stratification, and clinical decision-making.

**Key Findings**

• Symmetric Projection Attractor Reconstruction (SPAR) provides a visual distinction between the normal sinus rhythm electrocardiograms (ECGs) of subjects with a previous paroxysmal atrial fibrillation (PAF) diagnosis and those without.
• Machine learning using SPAR features, which quantify the morphology and variability of the signals, gave a cross-validated accuracy of 89.0% and an area under the receiver operating characteristic curve of 0.98 when discriminating between the PAF and non-PAF diagnoses.
• Our final machine learning model had excellent classification power for PAF, with a sensitivity of 94.8% and specificity of 87.1%.
• Our SPAR and machine learning methodology is easily implemented and uses only short ECG segments, supporting its incorporation into the monitoring and risk stratification of equine subjects for PAF.

## Introduction

Atrial fibrillation (AF) is a common cardiac arrhythmia in both human and equine populations. In humans, AF is associated with a decreased athletic performance^1^ and an increased risk of sudden cardiac death, stroke, and other coronary heart disease deaths.^2^ A decreased performance of equine athletes has also been associated with AF.^3^, ^4^, ^5^, ^6^ In an otherwise healthy horse, performance generally improves if normal sinus rhythm can be regained, although recurring AF episodes and other cardiac abnormalities give a much poorer prognosis.^3^^,^^4^ Furthermore, there are indications that, as in humans, equine AF episodes may be associated with collapse and sudden death.^7^^,^^8^

AF in both human and equine populations is diagnosed through the examination of electrocardiogram (ECG) traces; normal sinus rhythm is disrupted during an AF episode, presenting with irregular R-R intervals and an absent P wave, often replaced by small, rapid baseline undulations called “fibrillatory (f) waves.”^2^^,^^8^ Paroxysmal atrial fibrillation (PAF) is a type of AF where these intermittent arrhythmic episodes terminate spontaneously.^2^^,^^8^ In humans, PAF is believed to progress to sustained forms of AF, and has been indicated to carry an equivalent risk of stroke.^9^, ^10^, ^11^ While the underlying mechanisms for this are unclear, it supports the importance of the early detection of PAF. However, PAF is notoriously difficult to detect when the symptoms of AF have resolved and normal sinus rhythm has resumed. Longer-term, ambulatory monitoring is often used with the aim of capturing an AF event,^12^ although the robust analysis of large data volumes can be challenging.

Although the mechanism of AF is not fully known, a PAF diagnosis has been associated with pathophysiological changes, including left atrial enlargement and a loss of contractility, in both human^13^^,^^14^ and equine populations.^15^^,^^16^ These potential structural changes in the heart may result in subtle changes in the ECG, even once it has returned to sinus rhythm, and a number of recent studies using human data have suggested that the PAF diagnosis can be detected from such recordings.^17^^,^^18^ While this has been less considered in equine ECG, a diagnosis of PAF has been shown to be associated with a decreased sinus rhythm ECG complexity metric^19^ and can be classified using restitution analysis of the signals.^20^

Herein we present a preliminary study to discriminate between equine athletes with and without a diagnosis of PAF from normal sinus rhythm ECGs. The ECG data used in this work were obtained during periods of sinus rhythm in the subject to assess whether abnormalities could be detected in such baseline electrical signals that might reflect a predisposition to AF episodes. We investigated the indication of subtle differences in the signals by applying the recently developed Symmetric Projection Attractor Reconstruction (SPAR) method,^21^, ^22^, ^23^ which provides a novel and innovative means of visualizing and quantifying the shape and variability of an approximately periodic waveform, such as the ECG. The technique overcomes the challenge of detecting and extracting features directly from a signal, and uses all the available data to encapsulate the waveform morphology in a 2-dimensional image, while reducing the effect of baseline wander and being robust to signal outliers. Recent studies applying SPAR analysis to ECG signals include the detection of a genetic mutation associated with increased risk of arrhythmias in murine ECG,^24^ the capture of changes induced by dofetilide in human ECG,^25^ and the discrimination of sex in human ECG.^26^

## Methods

**Figure 1:**
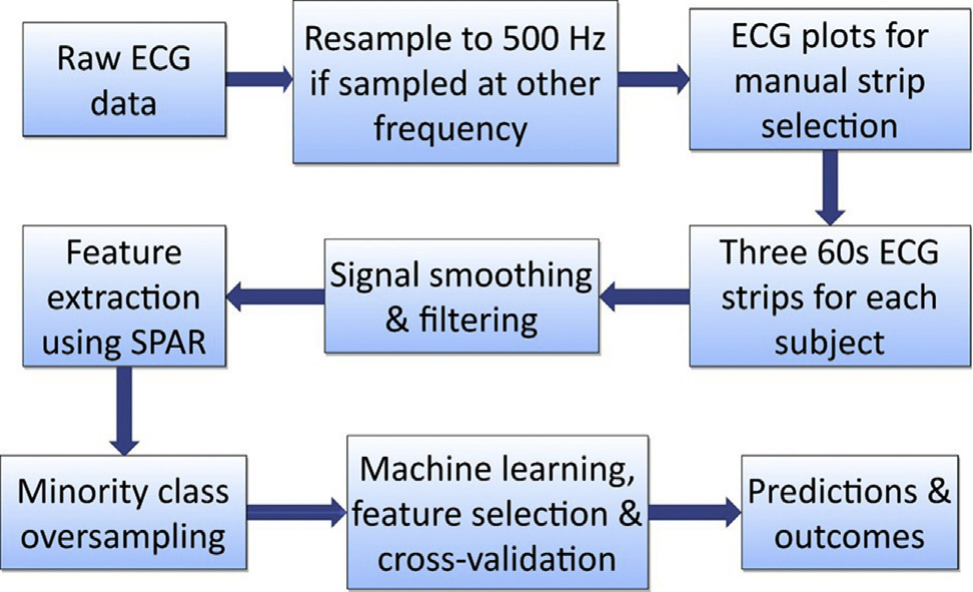
Flowchart of the overall methodology from the raw data to final classification. ECG = electrocardiogram; SPAR = Symmetric Projection Attractor Reconstruction.

We obtained a total of 139 ECG recordings from 19 equine athletes with a PAF diagnosis and 120 controls. After preprocessing of ECG strips of normal sinus rhythm, the SPAR method was applied to extract features of the ECG waveform shape and variability, and machine learning was employed to distinguish between horses with and without a diagnosis of PAF. An overview of the methodology is given in Figure 1, and further detail on all aspects of the method is provided in the Supplemental Material.

### Ethics statement

The electrocardiographic data used in this study were collected as part of routine clinical work in line with the Veterinary Surgeons Act. The study has also gone through the University of Surrey Non-Animal and Scientific Procedure Act (NASPA) ethical review self-assessment and based on the answers submitted, no further ethical review/approval is required, with local policy requiring sign-off by the Head of Department.

### Data

Data were obtained from 139 horses of racing age and in race training that presented for routine clinical work at Rossdales Equine Hospital and Diagnostic Centre (Newmarket, Suffolk, UK). The complete dataset consisted of 139 modified base-apex ECG recordings of equine athletes, split between 19 PAF subjects and 120 control subjects. PAF subjects were defined as those diagnosed with PAF by a specialist in equine internal medicine based on the presence of AF on previous ECG recordings, whereas those subjects in the control group had no known history of PAF episodes. ECGs were primarily recorded at rest, but also contained periods affected by environmental stimulus or exercise. The length of recording varied from 25 minutes to 48 hours, and we extracted a window from each signal of no more than 3 hours from the start of the recording to reduce unwanted change due to any diurnal effects.

Initially, ECGs were available for 91 of the 139 subjects (81 control and 10 PAF). These data were denoted dataset 1 and were used to develop the initial machine learning model (see Machine learning classification section below). Subsequently, the ECG recordings for the remaining 48 subjects (39 control and 9 PAF) were obtained and denoted dataset 2. This new set of data provided an independent test set that was used to perform an evaluation of the initial model performance, and then to generate a revised model. Finally, the 139 subjects were combined as dataset 3, which was used to develop a final machine learning model. Details of these 3 datasets are given in Table 1.

**Table 1:** Details of the 3 datasets, showing the number of subjects and number of electrocardiogram 20-second sub-strips obtained (the latter in brackets, and being 9 records per subject)

	Total size, subjects (sub-strips)	Control, subjects (sub-strips)	PAF, subjects (sub-strips)	Usage
Dataset 1	91 (819)	81 (729)	10 (90)	Training set for the initial model development
Dataset 2	48 (432)	39 (351)	9 (81)	Independent test set for the initial model evaluation Feature selection for revised model development
Dataset 3 *(being 1 and 2 combined)*	139 (1251)	120 (1080)	19 (171)	Cross-validation for final model development and evaluation

PAF = paroxysmal atrial fibrillation.

### ECG preprocessing

For each animal, 3 60-second strips of normal sinus rhythm with an acceptable signal quality (eg, clear R peaks and small baseline wander) were manually selected at an approximately equal spacing within a window of no more than 3 hours from the start of the recording. For some recordings, especially those with large sections of artefacts, it was necessary to take the 3 best available 60-second strips, which may not have been as widely separated.

Each 60-second strip was then split into 3 20-second sub-strips, giving a total of 9 records for each subject and 1251 records for 139 subjects overall. Although most of the strips selected were reasonably clean, there still existed baseline variation and high-frequency noise, and therefore filtering was applied as a final preprocessing step. The ECGs were filtered with an eighth-order high-pass Butterworth filter to remove low-frequency baseline variation, a zero-order Savitzky-Golay filter to smooth out sharp corners and edges, and a low-pass Parks-McClellan filter to remove high-frequency noise. Further details of the filtering method can be found in the Supplemental Material.

The filtered signals were then normalized by their respective 99.9 percentile amplitude values to remove amplitude disparity caused by different recording equipment.

### SPAR analysis

The SPAR method transforms the entirety of an approximately periodic signal into a corresponding 2-dimensional image, and the technique is well described in the original paper by Aston and colleagues.^21^ To apply the SPAR method, we place 3 equally spaced points on the signal. As these points move along the signal, we can generate a bounded representation of the whole waveform in 3-dimensional phase space. This 3-dimensional object is then projected to a 2-dimensional image, which we call an “attractor,” that presents with 3-fold symmetry, making it simpler to discern changes in the waveform morphology. Finally, a density is overlaid, highlighting the areas of the attractor image most frequently visited, and providing a visualization of waveform shape and variability that can be quantified by the features of the attractor density.

**Figure 2:**
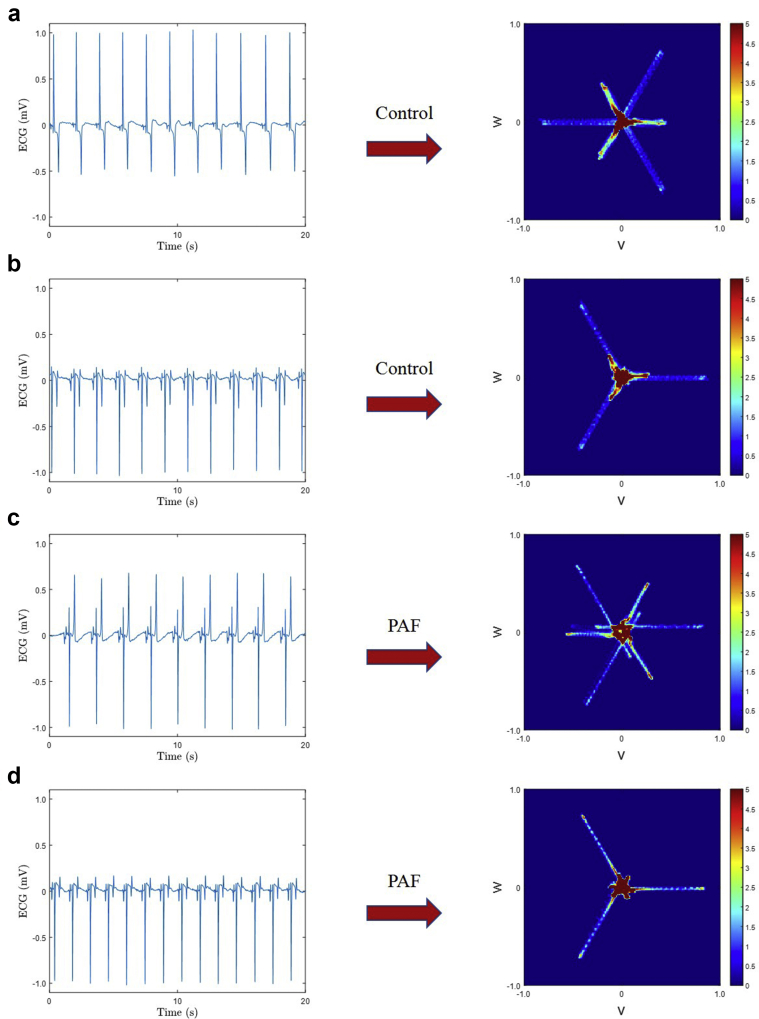
The 20-second electrocardiogram (ECG) sub-strip and its corresponding attractor for 2 different control subjects (**a, b**) and 2 different paroxysmal atrial fibrillation (PAF) subjects (**c, d**). Subtle differences in the rotation of the central core region of the attractor could be observed between the control and PAF groups for most subjects. This figure illustrates the differing appearance of equine ECGs and also that the attractor is independent of any vertical shift of the signal, since the resulting image remains centered on the origin of the (v,w) plane (see Aston and colleagues^21^ for further details).

For this study, we placed our 3 points on each 20-second ECG sub-strip, equally spaced by one-third of the average cardiac cycle length (average R-R interval, where the R peaks were determined by peak detection). We therefore generated an attractor for each 20-second sub-strip, which gave 9 attractors for each subject. A visual distinction could be observed between the attractors of the control and PAF groups for most subjects, which presented as a rotation of the central core region of the attractor, as illustrated in Figure 2 for 2 control and 2 PAF subjects. However, this difference was subtle, and we therefore chose to explore it further by applying machine learning techniques, which may also incorporate differences that could not be discerned visually in the attractor.

Each attractor image was quantified by taking 73 SPAR features that comprehensively captured its size, shape, symmetry, and density distribution over 7 regions (the core and 6 equal “arm” segments). These features included the maximum density of the attractor, the length of the arms, the angle of orientation of the arms, and the size of the central core region. In addition, we included the mean R-R interval of each strip as a further feature (giving 74 features in total), as the SPAR method provides a time normalization of the signal to concentrate on its morphology.

### Machine learning classification

We applied machine learning with the aim of classifying each 20-second ECG sub-strip as either control or PAF using the features drawn from the attractor. The results from each of the 9 sub-strips for a subject were then combined in a majority vote to classify the subject. Further details supporting our machine learning methodology can be found in the Supplemental Material and we restrict ourselves to an overview here.

The observed visual differences between control and PAF attractors were subtle and we sought to avoid overfitting to the data, so we chose to adapt an ensemble approach to classification of the 20-second sub-strips, combining the results from 3 machine learning classifiers. For these classifiers, we chose algorithms with fundamentally different approaches, taking a *k*-nearest neighbors (*k*-NN) algorithm as a “local” classifier and 2 support vector machine (SVM) algorithms with different kernels as “global” classifiers.^27^ For *k-*NN, we took *k* = 9 and a standardized Euclidean distance. The first SVM classifier applied a linear kernel with standardized features, while the second used a radial basis function (RBF) kernel without feature standardization, as noise in the features can be amplified by standardization and the nonlinear RBF kernel tends to overfit to this noise.

The SPAR features from each 20-second ECG sub-strip were input to each classifier, and the posterior probability scores (between 0 and 1) were obtained. Our ensemble model was then completed by a simple majority vote of the 3 classifiers (*k*-NN, SVM with linear kernel, and SVM with RBF kernel), giving a combined posterior probability for the likelihood of the 20-second sub-strip being PAF, with values ranging from 0 (control) to 1 (PAF).

Finally, the classification of a subject was determined as a majority vote of the 9 20-second sub-strips for the subject. The score for the majority vote was obtained as the number of records classified as PAF (between zero and 9) divided by the number of records (9), giving a value between 0 (control) and 1 (PAF). A score threshold of 0.5 between a classification of PAF or control was chosen to provide a simple majority vote.

#### Minority class oversampling

Owing to the natural prevalence of PAF, the PAF subjects were significantly under-represented compared with the controls, and this imbalance can significantly impact the performance of a machine learning classifier.^28^ We therefore applied an oversampling to increase the PAF class in the training data by generating synthetic data points, using the recently developed cluster-based Adaptive Semi-Unsupervised Weighted Oversampling method.^29^ Further details about this technique are given in the Supplemental Materials. We emphasize that our ensemble model was trained with data that included synthetic points, but we only classified the real data.

#### Cross-validation

As we only had data from a relatively small number of subjects, we assessed the performance of the machine learning model for both feature selection (see Feature selection below) and our final model using cross-validation, applying a modified Monte Carlo cross-validation technique,^30^ which we will term stratified repeated random sampling (SRRS). The SRRS method randomly allocated subjects to either the training or test set. The PAF records in the training set were then oversampled (as described in Minority class oversampling above), the ensemble model was trained, and a classification obtained for the test set subjects. This process was repeated a predefined number of times, enforcing unique combinations of the training set to avoid duplicate runs. We observed a decreasing absolute difference of the record and subject majority vote performance metric (see Performance metrics below) means between consecutive runs, suggesting that repeated SRRS provided a good indication of model performance.

When developing our initial model with the 91 subjects in dataset 1, we performed SRRS for feature selection (see Feature selection below) taking 74 control and 7 PAF subjects in the training set, with the remaining 7 control and 3 PAF in the test set. For our final model generated using the combined data in dataset 3 (139 subjects in total), we took 108 control and 15 PAF subjects for training and the remaining 12 control and 4 PAF for testing, and applied SRRS for both feature selection and evaluation of the final model.

#### Feature selection

We obtained 74 features from each 20-second ECG sub-strip by applying the SPAR method. It is likely that some of these features are less relevant for distinguishing PAF. Furthermore, machine learning algorithms can suffer from the “curse of dimensionality” and may perform better when fewer features are input.^31^ Therefore, we applied a sequential forward feature selection approach, weighted to emphasize the correct classification of PAF subjects, to determine an optimal reduced set of features.^32^ Details of the feature selection can be found in the Supplemental Material and in the Results below.

#### Performance metrics

We present a range of performance metrics^33^ for the classification of the 20-second sub-strips and the subjects to provide a broad summary of our results and allow comparison with other studies. In addition to accuracy, we also provide the sensitivity (true-positive rate), specificity (true-negative rate), and area under the receiver operating characteristic curve (ROC AUC), where we took PAF to be the positive class. Finally, we show the F_1_ scores for each of PAF and control. The F_1_ score is a result between 0 and 1, where a higher score indicates better precision and increased robustness.

## Results

### Feature selection and initial ensemble model training using dataset 1

For dataset 1 (91 subjects with 81 control and 10 PAF), 9 20-second ECG sub-strips were obtained for each subject, and the corresponding attractors were generated, from which we extracted 74 features. We then applied a forward feature selection (see Feature selection), which reduced this to an optimal set of 17 features. These included various statistical measures (mean, median, minimum, maximum, standard deviation, and quantiles) of the attractor symmetry, the length and density of the attractor arms, the size and density of the attractor core region, and the degree of rotation of the attractor arms from an “expected” position. These 17 features formed the input to an ensemble machine learning model, and an initial model was generated by training on all 91 subjects of dataset 1.

### Testing the initial model with dataset 2

**Figure 3:**
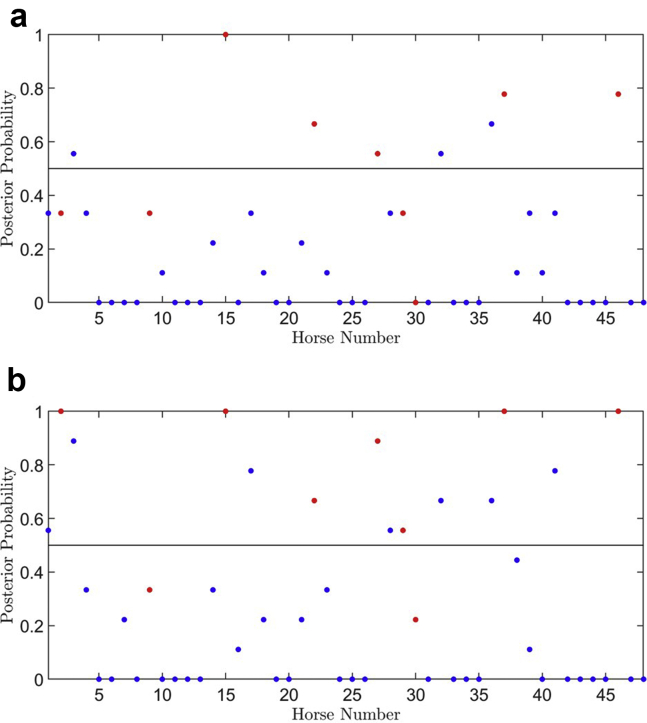
Posterior probability scores for likelihood of a paroxysmal atrial fibrillation (PAF) diagnosis by subject for the 48 subjects of dataset 2, with control subjects in blue and PAF subjects in red. **a:** Results for initial model trained using features derived from only dataset 1 (91 subjects). **b:** Results for revised model trained using features derived from the combined dataset 1 and dataset 2 (91 + 48 subjects), but trained on only dataset 1 data.

We were subsequently provided with a further 48 subjects (39 control, 9 PAF), which comprised dataset 2. This new set of data was collected under the same conditions and therefore provided an independent test set that could be used to perform a preliminary evaluation of the initial model. Figure 3a shows the posterior probability of PAF predicted by the model on these unseen subjects. The classification results for dataset 2 using the initial model (trained on dataset 1) were undiscriminating, with only 5 out of 9 PAF subjects correctly identified. The general performance of the model is shown in Table 2 under “Initial model.”

**Table 2:** Classification performance metrics for the initial, revised, and final machine learning models

Classification	Accuracy	Sensitivity (TPR)	Specificity (TNR)	ROC AUC	F_1_ PAF	F_1_ CTR
Initial model, features selected from dataset 1, trained on dataset 1, tested on dataset 2 (48 subjects)						
*Sub-strip*	81.3%	53.1%	87.8%	0.790	51.5%	88.4%
*Subject*	85.4%	55.6%	92.3%	0.863	58.8%	91.1%
Revised model, features selected from dataset 1 + dataset 2, trained on dataset 1, tested on dataset 2 (48 subjects)						
*Sub-strip*	80.1%	74.1%	81.5%	0.843	58.3%	86.9%
*Subject*	81.3%	77.8%	82.1%	0.907	60.9%	87.7%
Final model, features selected from dataset 3, SRRS cross-validated training and testing using dataset 3 (139 subjects)						
*Sub-strip*	87.9% (76.0% - 97.9%)	88.7% (66.6% - 100%)	87.6% (72.2% - 100%)	0.936 (0.829 - 0.998)	79.0% (62.2% - 95.6%)	91.4% (82.1% - 98.6%)
*Subject*	89.0% (75.0%–100%)	94.8% (75.0%–100%)	87.1% (66.7%–100%)	0.978 (0.896–1)	82.1% (60.0%–100%)	92.0% (80.0%–100%)

Sensitivity (true-positive rate, TPR), specificity (true-negative rate, TNR), and area under the receiver operating characteristic curve (ROC AUC) taking paroxysmal atrial fibrillation (PAF) as the positive class. The F_1_ scores are given for PAF and control subjects (F_1_ PAF and F_1_ CTR, respectively). Models assessed with stratified repeated random sampling (SRRS) cross-validation over 1000 runs show the mean result and the 95% coverage intervals (2.5th – 97.5th percentile range).

#### Revising the selected feature set

As the initial model was trained on only 91 subjects (dataset 1), it was suggested that this limited the choice of optimal features for PAF detection. As a test of this, we applied the forward feature selection process (see Feature selection) to the combined dataset of all 139 subjects (dataset 3), which resulted in a revised feature set of 14 features. While the features selected differed from the original set, the revised optimal feature set contained a similar spread and variety of attractor measures. However, it is difficult to draw any specific interpretation about individual features from this, since it is the combination of the subtle differences in weak predicting features rather than a single strong predicting feature that gives rise to accurate PAF detection.

We then trained a revised model on dataset 1 only (as before), but using the new optimal feature set of 14 features. As shown in Figure 3b, the resultant PAF detection rate went up to 7 out of 9 subjects and the posterior probabilities for all PAF subjects were increased (or the same for 1 subject), ie, most PAF subjects being more likely PAF by the model classification. A summary of the classification results is given in Table 2 under “Revised model,” supporting that the revised feature set improved the ability to classify PAF subjects correctly. While our test data are not wholly independent of the model in this case, owing to the inclusion of the data in the feature selection process, the revised model was still only trained on the original data, and we would anticipate that the features obtained from feature selection using only the training set would be more comprehensive and reliable as more equine data becomes available.

### A final cross-validated model with dataset 3

To assess the performance of the model reliably with the inclusion of the new data, we repeated the SRRS cross-validation technique on all the available data (dataset 3, 139 subjects with 139 × 9 = 1251 records), using the 14 features previously selected, as previously described (Revising the selected feature set). Over 1000 cross-validation runs, a mean accuracy of 89.0% (87.9%), a mean sensitivity of 94.8% (88.7%), and a mean specificity of 87.1% (87.6%) was achieved by subject (sub-strip), as shown in Table 2 under “Final run.” By including dataset 2 in the training set, we observed improvements in all metrics compared with the initial and revised models, except for a small decrease in specificity. In addition to this, the sensitivity is very high, which is expected given that we placed a larger emphasis on detection of PAF by assigning a heavier penalty on the sensitivity (true-positive rate) in the feature selection process (see Feature selection). Additionally, a mean ROC AUC of 0.978 was obtained, which further confirmed the excellent classification power of the final model. We again acknowledge that the cross-validated test sets are not wholly independent of the model, owing to using dataset 3 in the feature selection process. When more equine data become available, we would anticipate that the features selected using only the training set would be more representative and consistent, and a k-fold cross-validation incorporating both the feature selection and the model generation steps would allow us to comment further on the generalizability of the model.

**Figure 4:**
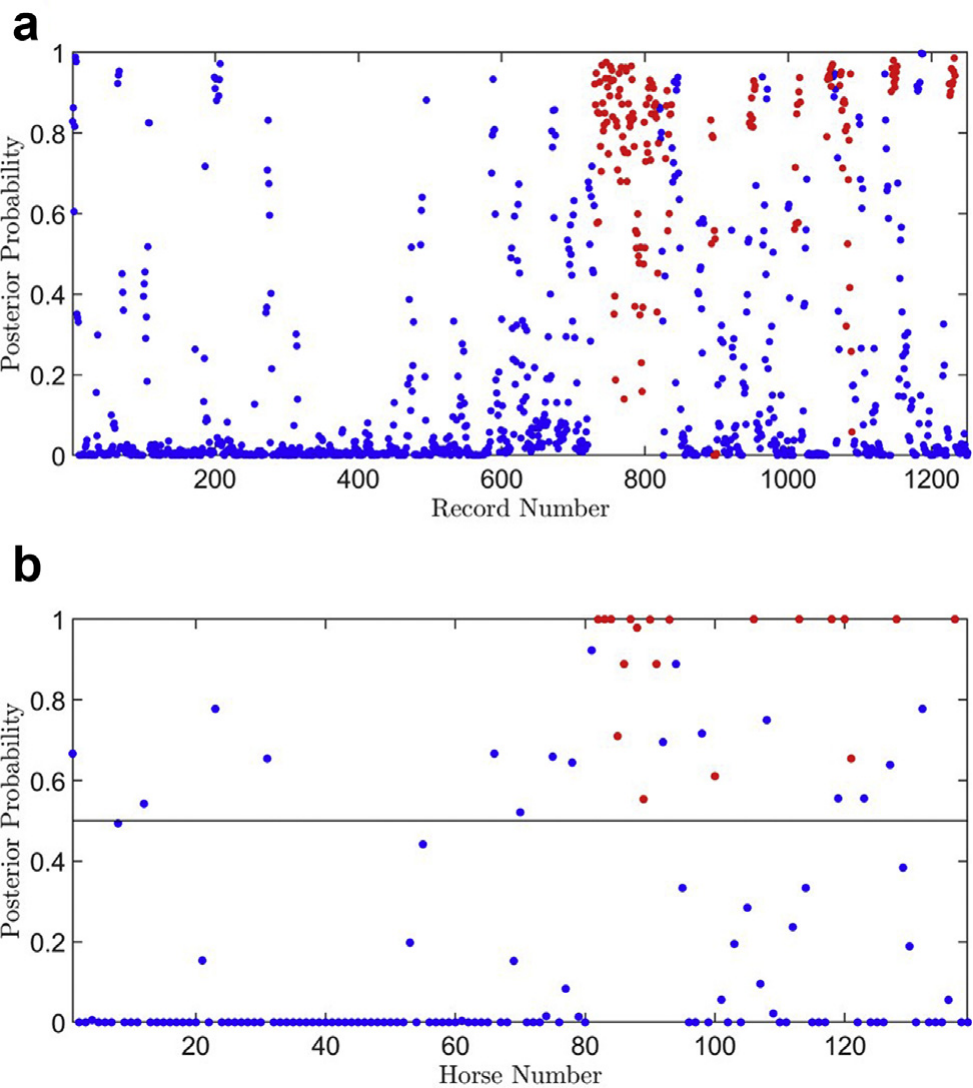
Mean posterior probability scores for likelihood of a paroxysmal atrial fibrillation (PAF) diagnosis over 1000 stratified repeated random sampling cross-validation runs for dataset 3, with control sub-strips/subjects in blue and PAF sub-strips/subjects in red. **a:** Classification of each 20-second sub-strip record (1251 sub-strips, 9 per subject). **b:** Classification of subject (139 subjects).

Figure 4 shows the mean posterior probability of PAF over 1000 SRRS cross-validation runs for each 20-second ECG sub-strip (Figure 4a) and subject (Figure 4b), and we observed that, on average, there is a clear difference between the control and PAF subjects. In Figure 4a, we observed that our voting classifier was able to detect a majority of the PAF sub-strips (red dots), as their posterior probabilities were generally closer to 1, compared with the control records (blue dots). This was even more pronounced in Figure 4b by subject, with all 19 PAF subjects being correctly classified and 103 out of 120 control subjects being correctly classified on average over the 1000 SRRS cross-validation runs, with the respective 95% coverage intervals given in Table 2 under “Final run.”

### SPAR density profiles

Despite the excellent predictive power of our final machine learning model, it may not be easy to explain and interpret how a classification was made. Therefore, we also examined the attractor images to support our understanding of how the machine learning model may distinguish between control and PAF subjects. A simple way to visualize an attractor image is by considering its polar coordinates and determining the density distribution in the radial (*r*) direction and the angular (θ) direction. The *r* density distribution can be further split into 2 regions, the high-density core at the center of the attractor and the lower-density attractor “arms.” Further details on the construction of a density profile can be found in Aston and colleagues^21^ and Lyle and colleagues.^26^

**Figure 5:**
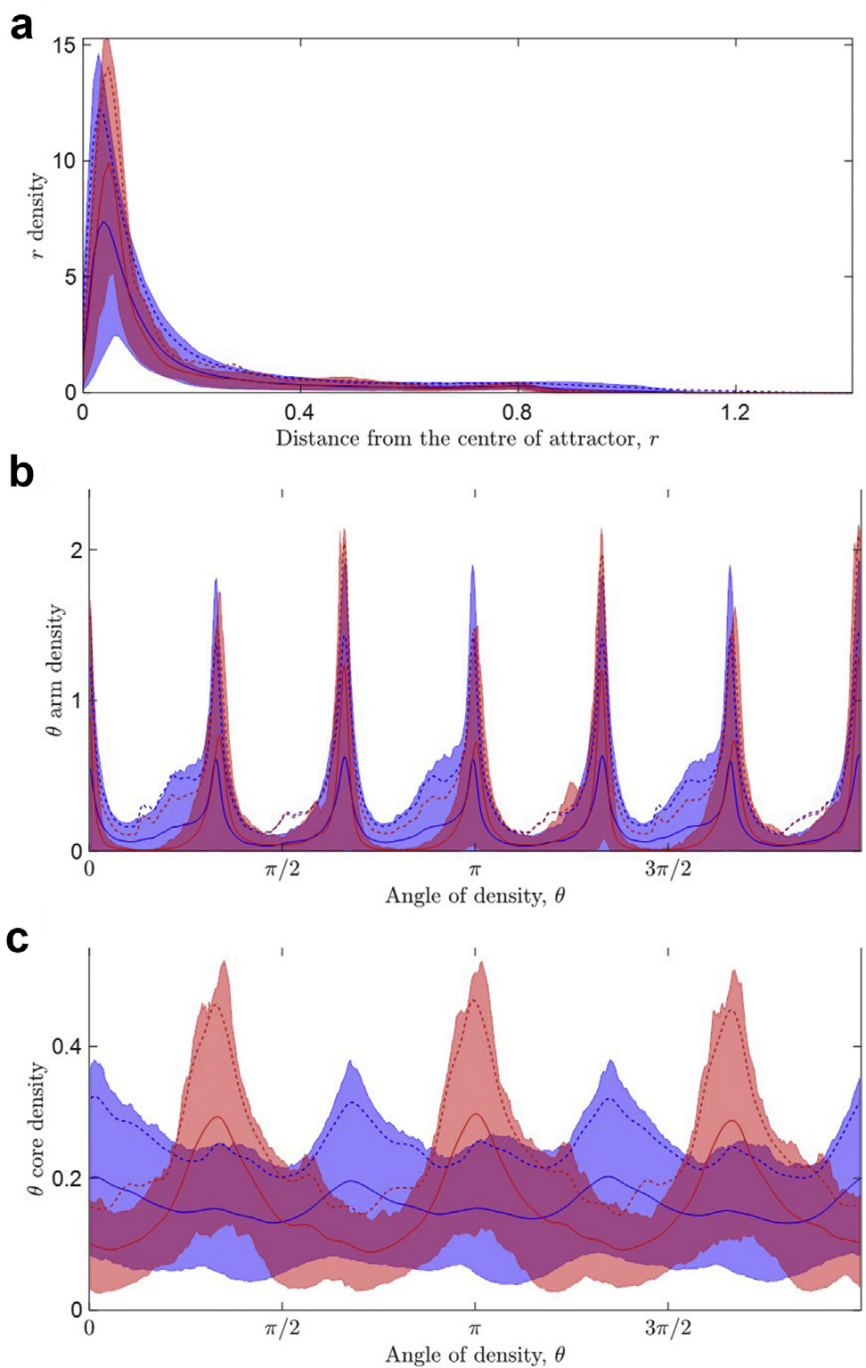
Density profiles for the attractors of dataset 3 (139 subjects, 1251 attractors) for (**a**) *r* density, (**b**) θ arm densities, (**c**) θ core densities, where *r* is the distance from the center of the attractor and θ reflects the angular movement around the attractor. Control records are shown in blue, paroxysmal atrial fibrillation records are shown in red. The solid lines correspond to the mean for each class of the data, the dotted lines correspond to the mean plus standard deviation, and the shaded regions indicate the data between the 5th and 95th percentiles.

The density profiles can also be used to visualize a number of attractors together, as shown in Figure 5 for all 139 subjects (1251 attractors from 1251 20-second sub-strips). Separating the control and PAF subjects by color, we observed clear differences in their respective densities. These plots are useful because we can visualize the mean distributions and the associated variations in the attractors for the control and PAF classes. We observed a higher average peak of the *r* densities for the PAF subjects in Figure 5a, along with taller peaks and slightly shifted peaks in the θ arm densities for PAF subjects in Figure 5b. Most strikingly, we noticed the taller peaks for the PAF subjects in terms of their θ core densities shown in Figure 5c. We note that the attractor arms are associated with the R peaks (which may be a positive or negative deflection in horses) in the ECG signal, whereas the core region is derived from the remainder of the signal and is clearly the more significant part of the attractor for this classification, as illustrated by comparing Figure 5b and Figure 5c.

#### Using the density profile to identify a data problem

**Figure 6:**
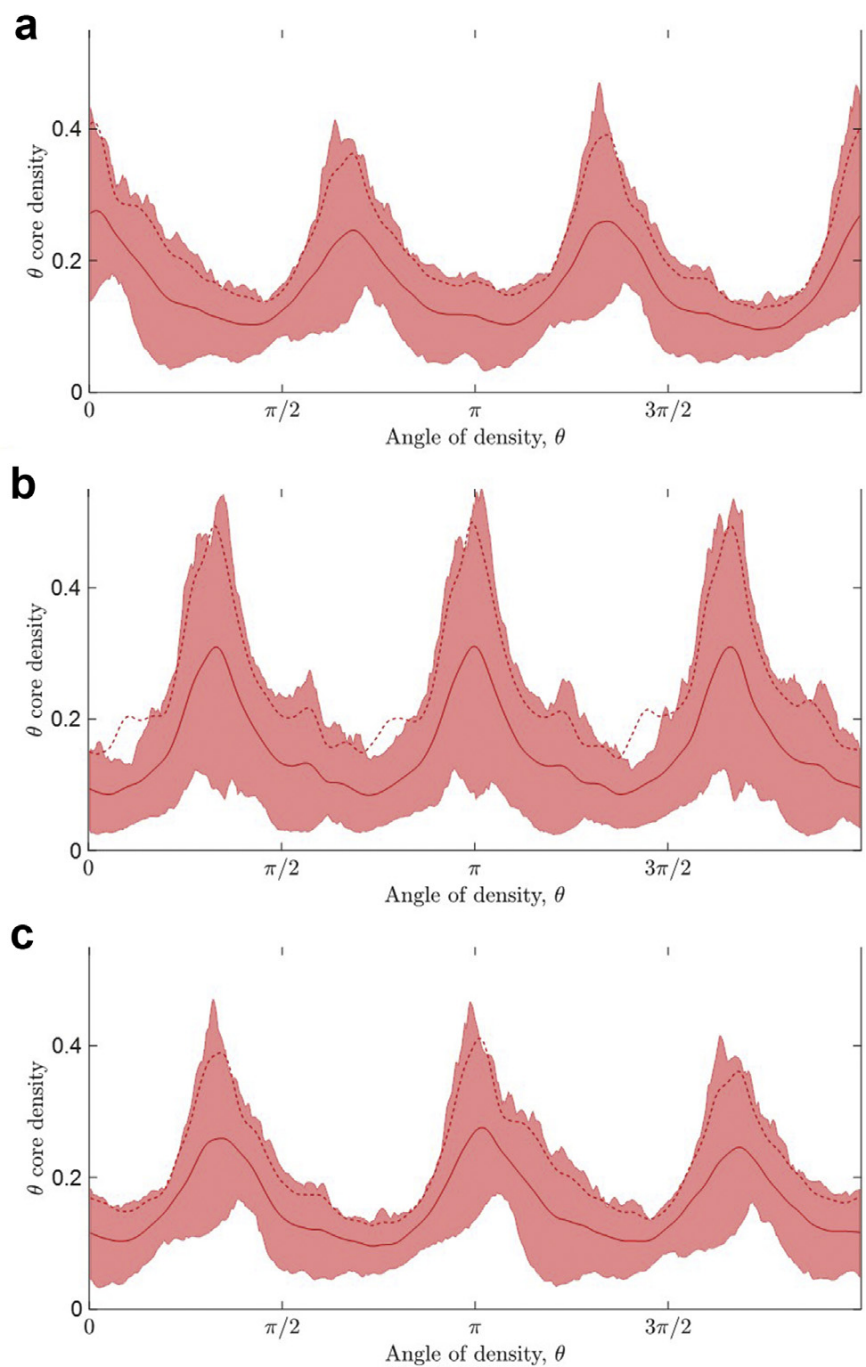
The θ core density profiles of the attractors for (**a**) the 6 paroxysmal atrial fibrillation (PAF) subjects with the (originally) 256 Hz electrocardiograms (ECGs), (**b**) the 13 PAF subjects with 500 Hz ECGs, and (**c**) the 6 PAF subjects with the (originally) 256 Hz ECGs after the signals were multiplied by -1. The solid lines correspond to the mean for each class of the data, the dotted lines correspond to the mean plus standard deviation, and the shaded regions indicate the data between the 5th and 95th percentiles.

The density profiles also played an important role in identifying a data problem with the ECG recordings for 6 PAF subjects in dataset 2, which were also the only records originally captured at 256 Hz (rather than 500 Hz). Initially, all 6 subjects were consistently misclassified with near 100% confidence by the model. However, when we reviewed the density profiles of the corresponding attractors, it was clear that θ core densities from these 6 subjects were out of phase with the θ core densities from the other 13 PAF subjects, as shown in Figure 6a and 6b, respectively. This indicated that the attractors of these 6 subjects were rotated by π (180°) from their expected presentation, corresponding to a difference in the signs of the signals between the (originally) 256 Hz and 500 Hz ECGs,^21^ which suggested that the misclassification of these subjects resulted from a difference in the recording equipment. Therefore, the 6 256 Hz ECG signals were multiplied by -1 before generating the attractors, which provided the expected density profiles, as illustrated in Figure 6c, and resulted in 5 out of the 6 subjects being correctly classified in the final model.

## Discussion

This preliminary study has shown that the SPAR method was able to discriminate between equine athletes with and without a PAF diagnosis, both visually in the attractors and their density profiles and quantitatively through generating machine learning models. Our final classifier had a high sensitivity, which is important, as it is more significant clinically to avoid misclassifying PAF cases compared with misclassifying controls, and indicated that it was possible to successfully detect PAF cases from short strips of (apparently) normal sinus rhythm ECG.

Traditional ECG analysis focuses on fiducial point identification and intervals based on the PQRST features of the signal. However, this gives a much reduced summary of the waveform and captures little information about its morphology. Under the premise that the discarded data may contain useful information about the health of a subject, we introduced the SPAR method, which uses all of the available waveform data and provides a unique visualization and quantification of its shape and variability. Furthermore, equine ECGs are often “noisier” than human ECGs, and the normal waveform is more variable (eg, the R peak may have a positive or negative deflection), making the robust detection of interval metrics challenging, whereas the SPAR method can still be applied.

Subtle differences could be visually discerned between PAF and control cases in the SPAR attractor image, and we elected to use machine learning on metrics drawn from the image to emphasize these. While the visual output of SPAR may indicate the use of deep learning on the attractor images, as Aston and colleagues^34^ and Venton and colleagues^35^ have demonstrated, we did not have enough data for such an approach, and therefore applied alternative machine learning algorithms using features extracted from the attractors. Furthermore, the 2 classes (PAF and control) were inherently imbalanced owing to the low prevalence of PAF, which we addressed with a synthetic oversampling of the PAF data, since machine learning on imbalanced classes can lead to poor performance. To reduce overfitting, we adapted an ensemble approach by taking the majority vote of 3 classifiers (*k*-NN, linear SVM, and SVM with RBF kernel) for each 20-second ECG sub-strip, with a further majority vote on the 9 ECG sub-strips for each subject to give a final classification.

Prior to machine learning, a sequential forward feature selection process was applied to identify an optimal feature set. Our final model selected 14 features, which were taken from across the attractor image, indicating that a number of weaker predictors were needed to encapsulate the subtle differences in the PAF subjects. The forward feature selection was made based on a metric that was weighted to ensure that greater emphasis was given to the correct classification of PAF subjects, and we observed that this was successful, as the sensitivity of the final model was high (94.8%). It would be useful to extend this by exploring different weightings in the selection metric to better understand the resulting sensitivity and specificity that are achieved by the subsequent model, as different monitoring applications or subject populations may benefit from an alternative balance of PAF and control classification rates.

The ECGs used in this study were of normal sinus rhythm, with subjects labeled as PAF cases owing to AF episodes observed on prior recordings. Thus we cannot be certain that all control labels were correct, since a horse may have had an earlier AF episode that was not captured. Although the PAF subject signals were obtained after the occurrence of any AF episodes, our ability to discriminate between PAF and control indicated that some difference remains in the ECG waveform, and that this can be encapsulated by the SPAR method. It would be useful to structure a further study that used ECG signals obtained prior to identified AF episodes to provide insight into whether subtle differences can precede the onset of a PAF diagnosis. Since the SPAR attractor is independent of the heart rate,^21^ it may also be helpful to incorporate complementary metrics that have been shown to be useful in the discrimination of AF episodes, such as R-R intervals^36^ or features drawn from the Poincaré plots of heart rate differences.^37^ Subtle changes in these metrics may also be distinguishable during normal sinus rhythm (distant from the actual AF episodes) and support the detection of the PAF diagnosis.

We selected 3 60-second ECG strips within a window of no more than 3 hours for each subject, since the ECG recordings were of different lengths and were frequently longer (up to and over 24 hours) for the PAF cases. Therefore, we had a consistent number of records for all subjects to avoid bias in the machine learning classification. However, we would expect that taking more records per subject would improve the model performance, and the use of more ECG strips, possibly spread over a longer window, should be explored, as this could inform the most appropriate ECG capture protocol for detecting PAF. If more data were obtained, or more ECG strips taken from each ECG recording, then a fully automated signal segment selection procedure based on determining both sinus rhythm and signal quality would be needed, as it would not be practical to review each strip.

The SPAR method provides a highly visual solution to the challenge of analyzing waveform shape and variability. The density profiles that provide one means of quantifying the attractor image can also be used directly as a tool to aid the interpretation of a machine learning classification, as we demonstrated in Figure 5. Interestingly, the density profiles also indicated a problem with the ECG data capture for 6 subjects, which allowed us to make a simple adjustment to rectify this.

While the detection of PAF from equine sinus rhythm ECGs has clinical value, it has not been widely explored as a classification problem until recently. Work has been undertaken on similar study populations using restitution,^20^ where the R-R, QT, and TQ intervals were taken as ECG features, and complexity analysis,^19^^,^^38^ where the ECG signal was converted to a sequence of binary numbers and its pattern analyzed for a degree of disorderliness (complexity). While the datasets were not identical, both studies showed that PAF subjects could be detected, with best ROC AUC of 0.90 and 0.95 from restitution^20^ and complexity^19^ analysis, respectively. Our comparable result in this study was a ROC AUC of 0.98 achieved by subjects in our final model, indicating that the SPAR method may provide supplementary analysis for this problem. Furthermore, both restitution and complexity analysis typically require low-heart-rate, resting ECGs, whereas SPAR is not restricted. Overall, the restitution and complexity studies used less than the 3 minutes (3 60-second strips) of data applied in this study, but SPAR was applied to individual 20-second sub-strips, which would be too short for the alternative techniques, and still achieved a ROC AUC of 0.94 as separate records. Our human ECG studies have typically used 10-second diagnostic ECGs,^25^^,^^26^^,^^39^ and extension of this study should evaluate a similar approach. We also propose that we should build on our ensemble machine learning approach using the 3 classifiers (*k*-NN, linear SVM, and SVM with RBF kernel) by incorporating features drawn from the 3 techniques (SPAR, restitution, and complexity) to assess their complementary attributes in the classification of PAF.

This article has presented a further successful application of the SPAR analysis of ECG signals, supporting that SPAR can extract more nuanced information from a normal sinus rhythm waveform to distinguish between horses with and without a diagnosis of PAF. While our study focused on retrospective equine data, there has been a recent surge of interest in both the retrospective detection and prospective prediction of PAF in human sinus rhythm ECGs, driven by clinical need, and supported by larger labeled datasets, advances in machine learning techniques, and more powerful hardware. Notably, Attia and colleagues^40^ obtained more than 180,000 adult ECG recordings and achieved an accuracy of 79.4% (ROC AUC of 0.87) for a single 10-second, 12-lead recording extracted up to 31 days before a recorded AF (or atrial flutter) ECG, which rose to 83.3% when all available sinus rhythm recordings from the 31 days were used. Alexeenko and colleagues^41^ extended the complexity analysis from equine ECGs^19^ to a pilot study using repeated 28-second single-lead sinus rhythm ECG from 52 older adult subjects, and discriminated PAF patients with a ROC AUC of 0.92, indicating the earlier methodology applied to equine ECG (ROC AUC of 0.95) was appropriate for the analysis of human ECG data. We recommend that a similar study be undertaken to extend the detection of PAF using the SPAR method to larger volumes of human data.

## Conclusion

The SPAR method is a novel and intuitive means of encapsulating the morphology and variability of an approximately periodic signal,^21^ and we have demonstrated that it is simple to apply to normal sinus rhythm equine ECG data of varying heart rates, generating attractor images that capture subtle waveform differences and can be easily quantified. Coupling this quantification with an ensemble machine learning approach enabled us to develop a model with excellent classification power for PAF, while the SPAR density profiles provided further insight into how this classification was achieved. Our results appeared to be complementary to similar analysis on equine ECG,^19^^,^^20^ and we now aim to extend this to a larger study using human data.

While the ability to both detect PAF retrospectively and predict a subsequent AF episode from apparently normal sinus rhythm ECG is an important problem when managing equine athletes, it has an even greater relevance in human populations, where it has been associated with various clinically adverse events, including death.^2^ Our simple and successful implementation of SPAR in this preliminary study indicates that the SPAR method is an ideal candidate to incorporate into the monitoring and risk stratification of human patients to support clinical decision-making.
