# Supplementary material for: Detecting paroxysmal atrial fibrillation from normal sinus rhythm in equine athletes using Symmetric Projection Attractor Reconstruction and machine learning
Source: Cardiovasc Digit Health J. 2022 Feb 14;3(2):96–106. doi: 10.1016/j.cvdhj.2022.02.001 (PMC9043370; doi:10.1016/j.cvdhj.2022.02.001)
Supplement: Supplemental Material [file mmc1.docx]

**Supplementary Material**

This Supplementary Material provides further information for the Methods. Some text from the main paper is repeated here intentionally for ease of reading.

# ECG collection and data

133 ECGs were recorded using a Televet 100 recorder (Engel Engineering Services GmbH, Germany), which has signal bandwidth of 0.05 - 100 Hz and sampling rate of 500 Hz, while the remaining six ECGs (all PAF) were recorded using a Lifecard CF recorder (Spacelabs Healthcare, USA), which has a sampling rate of 256 Hz. The six 256 Hz signals were resampled to 500 Hz using linear interpolation for consistency.

The length of recording varied from 25 minutes to 48 hours, and we extracted a window from each signal of no more than three hours from the start of the recording to reduce unwanted change to diurnal effects. Horses in the PAF group had longer recordings in general, with a median length of 17.4 hours in the PAF group, compared with 1.2 hours in the control group.

# ECG preprocessing

Each 20-second sub-strip of ECG was filtered independently, with the cut-off frequencies of the filters determined by the frequency response within the sub-strip. The rationale behind this approach was to ensure consistency for every record, e.g. large noise in one segment of the signal would have minimal impact on the filter performance for other ECG strips. Before the filtering process, each ECG interval was first extended by appending its flipped signal to the start and the end, in order to reduce the inaccuracies caused by the filters near the two end points and to aid implementing phase shift correction for the lag caused by the filtering process.

The following filters were applied:

1. an 8^th^ order high-pass Butterworth filter^1^ to remove low frequency baseline variation, with a cut-off frequency set by the first frequency with a value above 0.5 Hz that corresponds to a response peak which has a minimum normalised prominence of 0.02 in the frequency domain minus 0.2 Hz, with a maximum value of 0.7 Hz;
2. a 0^th^ order Savitzky-Golay filter^2^ to smooth out sharp corners and edges; and
3. a low-pass Parks-McClellan filter^1^ to remove high frequency noise, with a cut-off frequency set by the last frequency that corresponds to the response peak which has a minimum normalised prominence of 0.02 in the frequency domain plus 0.2 Hz, with a minimum value of 12.2 Hz and a transition band of 50 Hz in width.

The filtered signals were normalised by their respective 99.9 percentile amplitude values to remove amplitude disparity caused by different recording equipment.

# Machine learning classification

The three machine classifiers^3^, *k*-NN, SVM with linear kernel and SVM with RBF kernel, selected for the ensemble model balanced ‘local’ and ‘global’ classification, and were implemented in Matlab R2020b. In each case, we used the default parameters for each classifier, other than the following, which gave the best results in preliminary work.

1. For *k-*NN, we took $k=9$ and a standardised Euclidean distance,
2. For linear SVM, we applied standardised features (features normalised by their corresponding weighted column means and standard deviations) to prevent features with large values from being dominant during the training process.
3. For SVM with a radial basis function (RBF) kernel, features were not standardised, as this can amplify noise in the features and the nonlinear RBF kernel tends to overfit to this noise.

Additionally, we chose the expected proportion of outliers to be 1% based on empirical knowledge.

# Minority class oversampling of SPAR features

Due to the natural prevalence of PAF, the PAF subjects were significantly under-represented compared with the controls, and this imbalance can significantly impact the performance of a machine learning classifier, since most standard machine learning algorithms expect an approximately equal amount of data from each class. We used Adaptive Semi-Unsupervised Weighted Oversampling (A-SUWO) a recently developed cluster-based oversampling technique which oversamples the minority class data to match with the majority class, to generate synthetic minority data points.^4^

Compared to previous methods such as SMOTE,^5^ Borderline SMOTE,^6^ Safe-Level SMOTE^7^ and ADASYN^8^, A-SUWO improves on the ability to generate synthetic minority class data near the boundaries to the majority class, which are often hard to learn by a machine learning classifier, while avoiding generating minority data that overlap with the majority class. In contrast with the conventional unsupervised clustering method, A-SUWO uses information from the majority class (hence semi-unsupervised) to determine the clusters and the sizes of the oversampled minority clusters before the oversampling stage. For A-SUWO, a synthetic instance is generated between the original point and one of its neighbours if they belong to the same minority cluster. Additionally, synthetic points are generated in all minority clusters to avoid the classifier being biased towards the oversampled clusters.

For our problem, the prior probabilities of each class for the machine learning classifiers are set to be 50%, i.e. the expected occurrences of both classes are equally likely, after the classes were balanced using A-SUWO, as shown in Supplement Table 1. We emphasise that the ensemble model was trained by including the synthetic points in the training set, but a classification was only made for the real data.

# Feature selection

We obtained 74 features from each 20-second ECG sub-strip by applying the SPAR method. It is likely that some of these features are less relevant for distinguishing PAF. Furthermore, machine learning algorithms can suffer from the ‘curse of dimensionality’, and may perform better when fewer features are input.^9^ Therefore, we applied a sequential forward feature selection approach^10^, weighted to emphasise the correct classification of PAF subjects, to determine an optimal reduced set of features.

Starting with an empty feature set, the sequential forward feature selection is an iterative feature selection method that successively adds the feature to the existing feature set which gives the best result, until there is no further improvement.^10^ It greatly increases the chance of obtaining the optimal feature set (i.e. a set of features that produces the best result) whilst exploring only a small subset of the whole feature combination space.

We modified the sequential forward feature selection method such that the selection process terminated after a fixed number of iterations, which was done to ensure a global minimum is reached within a specified range of feature set size. The maximum feature set size was chosen to be 20, since we observed that the performance plateaued and started to decline after this.

There are many scores that could be used for evaluating the performance of the model for the feature selection. We defined PAF to be the positive class and chose the score

$$y=1-\left( \frac{(3\times TP{R)}^{-1}+TNR^{-1}+PPV^{-1}+NPV^{-1}}{4} \right)^{-1},$$

which is one minus the weighted harmonic mean of the true positive rate (sensitivity, TPR), true negative rate (specificity, TNR), positive predictive value (PPV) and negative predictive value (NPV).^11^ A weighting factor of 3 is placed on the true positive rate to create a model that emphasises the correct classification of PAF. The harmonic mean is useful because it penalises the final score heavily if any of the measures have low values.

As we only had data from a relatively small number of subjects, we applied a modified Monte Carlo cross-validation technique^12^ which we called stratified repeated random sampling (SRRS), both for feature selection and when generating our final model. The SRRS method randomly allocated subjects to either the training or test set. The PAF records in the training set were then oversampled (as described in Supplement section 4), the ensemble model was trained, and the feature selection score obtained for the test set subjects.

The feature selection score was calculated by averaging the results for the SRRS cross-validation test sets, with number of the cross-validation runs was set to 100 to allow for a realistic run time (rather than the 1,000 runs used for final model generation). Once the scores were obtained, the top two feature sets were used in a final SRRS cross-validation with 1,000 runs such that the feature set which produced the highest accuracy and TPR was chosen as the optimal feature set. This last step was implemented to increase the chance of finding an optimal feature set and to reduce any influence from randomness.

# References

1. Parks TW, Burrus CS: Digital Filter Design. John Wiley \& Sons, 1987,.

2. Schafer RW: What is a Savitzky-Golay filter? [Lecture notes]. IEEE Signal Process Mag IEEE, 2011; 28:111–117.

3. Hastie T, Tibshirani R, Friedman J: The Elements of Statistical Learning. 2nd Edition. Springer, 2008,.

4. Nekooeimehr I, Lai-Yuen SK: Adaptive semi-unsupervised weighted oversampling (A-SUWO) for imbalanced datasets. Expert Syst Appl 2016; 46:405–416.

5. Chawla N V., Bowyer KW, Hall LO, Kegelmeyer WP: SMOTE: Synthetic Minority Over-sampling Technique. J Artif Intell Res 2002; 16:321–357.

6. Han H, Wang W-Y, Mao B-H: Borderline-SMOTE: A new over-sampling method in imbalanced data sets learning. Adv Intell Comput Springer Berlin Heidelberg, 2005, pp. 878–887.

7. Bunkhumpornpat C, Sinapiromsaran K, Lursinsap C: Safe-level-SMOTE: Safe-level-synthetic minority over-sampling technique for handling the class imbalanced problem. Pacific-Asia Conf Knowl Discov Data Min Springer Berlin Heidelberg, 2009, pp. 475–482.

8. He H, Bai Y, Garcia EA, Li S: ADASYN: Adaptive synthetic sampling approach for imbalanced learning. 2008 IEEE Int Jt Conf Neural Networks 2008, pp. 1322–1328.

9. Blum AL, Langley P: Selection of relevant features and examples in machine learning. Artif Intell 1997; 97:245–271.

10. Guyon I, Elisseeff A: An introduction to variable and feature selection. J Mach Learn Res 2003; 3:1157–1182.

11. Powers DMW: Evaluation: From precision, recall and F-factor to ROC, informedness, markedness and correlation. J Mach Learn Technol 2011; 2:37–63.

12. Kuhn M, Johnson K: Over-fitting and model tuning. Applied Predictive Modeling. Springer, 2016, pp. 61–92.

# Supplement tables

Supplement Table 1 Number of subjects (sub-strips) in the training data for the controls and PAF prior to applying the synthetic oversampling procedure using A-SUWO, with the number of PAF sub-strips after oversampling. The median and 2.5th to 97.5th percentile range is given for the SRRS cross-validations as the oversampling method was applied to each of the 1,000 cross-validation runs.

| **Training data** | **Number of controls**  subject (sub-strips) | **Number of original PAF**  subject (sub-strips) | | **Number of oversampled PAF**  median sub-strips  (2.5^th^ to 97.5^th^ percentile) | |
| --- | --- | --- | --- | --- | --- |
|  | | | | | |
| Initial model, SRRS cross-validated training and testing using Dataset 1 (91 subjects) | | | | | |
|  | 74 (666) | | 7 (63) | | 662 (659 – 664) |
|  | | | | | |
| Initial model and revised model, model trained on all Dataset 1 (91 subjects) | | | | | |
|  | 81 (729) | 10 (90) | | 722 | |
|  | | | | | |
| Final model, SRRS cross-validated training and testing using Dataset 3 (139 subjects) | | | | | |
|  | 108 (972) | 15 (135) | | 961 (957 – 966) | |
